# Hepatitis C virus genotype and subtype distribution in Chinese chronic hepatitis C patients: nationwide spread of HCV genotypes 3 and 6

**DOI:** 10.1186/s12985-015-0341-1

**Published:** 2015-07-25

**Authors:** Wei Ju, Song Yang, Shenghu Feng, Qi Wang, Shunai Liu, Huichun Xing, Wen Xie, Liying Zhu, Jun Cheng

**Affiliations:** Institute of Infectious Diseases, Beijing Ditan Hospital, Capital Medical University, 8 East Jingshun Street, Chaoyang District Beijing, 100015 China; Center of Hepatology, Beijing Ditan Hospital, Capital Medical University, 8 East Jingshun Street, Chaoyang District Beijing, 100015 China; Department of Infectious Diseases, The Fourth Hospital of Harbin Medical University, 37 Yiyuan Street, Nangang District Harbin, 150081 China

**Keywords:** Hepatitis C virus (HCV), Genotype, Phylogenetic analysis

## Abstract

**Background:**

Hepatitis C virus (HCV) genotype and subtype are related to disease progression and response to antiviral therapy. Current HCV genotype and subtype distribution data, especially for genotypes 3 and 6, are limited in China. Our purpose was to investigate the current HCV genotype and subtype distributions in chronic hepatitis C patients in China.

**Methods:**

Chronic hepatitis C patients (*n* = 1012) were enrolled, and demographic information and possible transmission risk factors were collected. Serum samples were subjected to reverse-transcription polymerase chain reaction, followed by direct DNA sequencing and phylogenetic analysis of the NS5B and core/E1 regions to determine HCV genotypes/subtypes. The geographical distributions of HCV genotypes/subtypes were analyzed. Demographic information and transmission risk factors were compared between different HCV genotypes/subtypes.

**Results:**

Four genotypes and seven subtypes of HCV were detected in 970 patients. Subtypes 1b, 2a, 3a, 6a, 3b, 6n, and 1a were detected at frequencies of 71.96 %, 19.90 %, 3.20 %, 2.16 %, 1.96 %, 0.41 %, and 0.41 %, respectively. Genotypes 3 and 6 showed an increasingly wide geographic distribution over time. Patients with subtypes 1b and 2a were older than those with 3a, 3b, 6a, and 6n subtypes (*p* < 0.05 in all subtypes). More genotype 1 and 2 patients underwent blood transfusion than those with genotype 3 (all *p* < 0.05). More genotype 3 and 6 patients had a history of intravenous drug use than those with genotypes 1 and 2 (all *p* < 0.05).

**Conclusions:**

Though subtypes 1b and 2a are still the most prevalent HCV subtypes in China, genotype 3 and 6 HCV infections have already spread nationwide from southern and western China.

**Electronic supplementary material:**

The online version of this article (doi:10.1186/s12985-015-0341-1) contains supplementary material, which is available to authorized users.

## Background

Hepatitis C virus (HCV) is a leading cause of chronic liver disease and presents a major threat to global public health. Worldwide, more than 185 million people have been infected [1], and these individuals face an increased risk of developing liver cirrhosis and hepatocellular carcinoma.

HCV can be classified into seven genotypes and at least 67 confirmed subtypes, 20 provisionally assigned subtypes, and 21 unassigned subtypes [2]. Genotypes 1, 2, and 3 are distributed globally. In contrast, genotypes 4, 5, and 6 are more concentrated to specific regions. Genotype 4 and subtype 5a are mainly found in Middle Eastern countries and the northern part of South Africa, respectively. Genotype 6 is mainly found in China and Southeast Asia, especially in the Hong Kong Special Administrative Region of the People's Republic of China (HKSAR), Thailand, and Vietnam [3, 4].

In China, the estimated prevalence of HCV is 0.43 % [5]. A previous report indicated that subtypes 1b and 2a were the dominant subtypes in China, although the incidences of genotype 3 and 6 HCV infections were increasing [6]. Owing to the changing routes of transmission and increasing global travel, the geographical and genetic diversity of HCV is constantly changing. Recently, studies on HCV genotype distribution in local regions and certain patients populations were reported [7–10]. Yet nationwide HCV genotype and subtype distribution data of chronic hepatitis C patients is limited. We conducted this large-sample, multi-centered study to investigate the status quo of HCV genotype and subtype distribution in chronic hepatitis C patients in China.

## Results

### Characteristics of the study population

Overall, 1012 chronic hepatitis C patients were enrolled from 23 centers. Patients were located in 24 administrative units from six different geographic regions throughout China. Table 1 summarizes the demographic features and geographic origins of all enrolled patients.

**Table 1:** Demographic features and geographic origins of 1012 patients enrolled in the study

Characteristic	Patients
Gender, n (%)	
Male	506 (50.0)
Female	506 (50.0)
Age, years	
Median (range)	46 (12–99)
Mean ± SD	48.8 ± 14.7
Geographic origin, n (%)	
Northeastern China	111 (10.97)
Northern China	403 (39.82)
Central China	40 (3.95)
Eastern China	275 (27.17)
Southern China	59 (5.83)
Western China	124 (12.25)

### HCV sequence amplification, genotyping, and phylogenetic analysis

Reliable nucleotide sequences of the NS5B and core/E1 region were obtained for 970 samples and used to construct phylogenetic trees. The phylogenetic analysis of both the NS5B and core/E1 regions revealed the presence of genotypes 1, 2, 3, and 6, while no genotype 4, 5, or 7 strains were found in this study (Table 2).

**Table 2:** Geographical distributions of HCV subtypes of 1012 patients

Geographic origin	n (%)	HCV subtypes							
		1a	1b	2a	3a	3b	6a	6n	NA^a^
Northeastern China	111(10.97)	0	71	37	0	1	1	0	1
Northern China	403(39.82)	1	270	90	13	7	3	0	19
Eastern China	275(27.17)	3	212	23	10	6	8	3	10
Central China	40(3.95)	0	31	8	0	0	0	0	1
Southern China	59(5.83)	0	36	9	4	1	9	0	0
Western China	124(12.25)	0	78	26	4	4	0	1	11
Total	1012(100)	4	698	193	31	19	21	4	42

^a^HCV RNA could not be amplified for genotyping

HCV genotype 1 was the most prevalent, found in 702 (72.37 %) patients. The next most abundant genotypes were 2, 3, and 6, found in 193 (19.90 %), 50 (5.15 %), and 25 (2.58 %) patients, respectively. Evaluations of subtype revealed that the most common was 1b, accounting for 71.96 % (698/970) of cases, followed by 2a (19.90 %; 193/970). Subtypes 3a, 6a, and 3b were identified in 31 (3.20 %), 21 (2.16 %), and 19 (1.96 %) patients, respectively. The least frequent subtypes were 1a and 6n, with each accounting for 4 (0.41 %) specimens.

In phylogenetic analyses of the NS5B and core/E1 regions, the sequences of each subtype were randomly distributed with other corresponding reference sequences derived from the Los Alamos HCV database. No clear geographical clustering of Chinese isolates in this study was detected (Additional files 1, 2, 3, 4, 5, 6 and 7).

### Geographic distribution of HCV subtypes in China

Subtypes 1b and 2a were detected in all 6 geographic regions. Subtype 3b was found in all geographic regions except central China. Subtype 3a was found in 4 geographic regions, but not in central or northeastern China. Subtype 6a was found in 4 geographic regions but not in central or western China. Subtype 1a was isolated from patients in eastern and northern China. Subtype 6n strains were isolated from subjects from eastern and western China. All 7 subtypes were detected in eastern China. Other subtypes were found in northern (subtype 6), southern and western (subtype 5), and northeastern (subtype 4) China. Only 2 subtypes were detected in central China.

### Age versus subtype in the patient population

Age differences were observed for some HCV subtypes. Patients infected with HCV subtype 1b (49.8 ± 15.0 years) and 2a (51.2 ± 12.9 years) were older than those infected with 3a (36.1 ± 9.8 years), 3b (37.0 ± 9.2 years), 6a (38.2 ± 12.1 years), and 6n (31.5 ± 10.3 years; *p* < 0.05 in each for all pairwise comparisons of 1b and 2a with 3a, 3b, 6a, and 6n). There were no differences in the mean ages between subtypes 1b and 2a (all *p >* 0.05). There was also no difference in the mean ages between patients with subtypes 3a, 3b, 6a, and 6n (*p >* 0.05 in all cases).

### HCV transmission risk factors and different genotypes

A comparison of patients’ risk factors for HCV infection and the detected HCV subtypes are shown in Table 3. A greater proportion of genotype 1 (*p* = 0.002) and 2 (*p* = 0.011) patients underwent blood transfusion compared to patients with genotype 3 infections. More genotype 1 patients underwent surgery than those with genotype 3 (*p* = 0.003). More genotype 2 patients underwent invasive procedures than those with genotype 1 (*p* = 0.015). Invasive procedures were performed more frequently in patients with genotype 2 and 3 infections compared to genotype 6 (all *p* < 0.05). The history of IDU was more prevalent in genotype 3 and 6 patients than in patients with genotypes 1 or 2 (all *p* < 0.05).

**Table 3:** Reported HCV transmission risk factors associated with different HCV genotypes

HCV genotype	Blood transfusion, n (%)	Surgery,n (%)	Invasive Procedure, n (%)	IDU, n (%)	Family history, n (%)
1 (*n* = 702)	265 (37.75)	262 (37.32)	105 (14.96)	22 (3.13)	13 (1.85)
2 (*n* = 193)	67 (34.72)	64 (33.16)	43 (22.28)^c^	2 (1.03)	3 (1.55)
3 (*n* = 50)	8 (16.0)^a,b^	11 (22.0)^a^	11 (22.0)	10 (20.0)^a,b^	0 (0)
6 (*n* = 25)	4 (16.0)	6 (24.0)	1 (4.0)^d,e^	5 (20.0)^d,f^	0 (0)

^a^Genotype 1 vs. genotype 3: *p* < 0.05; ^b^genotype 2 vs. genotype 3: *p* < 0.05; ^c^genotype 1 vs. genotype 2: *p* < 0.05; ^d^genotype 2 vs. genotype 4: *p* < 0.05; ^e^genotype 3 vs. genotype 4: *p* < 0.05; ^f^genotype 1 vs. genotype 4: *p* < 0.05; IDU, intravenous drug use

Family clustering of HCV was reported in 16 patients with subtypes 1b (*n* = 13) and 2a (*n* = 3), although these patients were all from different families. The mean age was 50.1 ± 10.9 (range, 21–65) years and 37.50 % (6/16) of the patients were female. They were from all geographic regions, except Southern China.

## Discussion

Knowledge on HCV genotypes and subtypes is important for clinical management of chronic hepatitis C, since discrete HCV genotypes respond differently to several treatment regimens [11, 12]. Treatment with pegylated interferon (IFN) and ribavirin is still the standard of care for chronic hepatitis C patients in China. However, several direct-acting antivirals (DAA) are in Phase III clinical trials. Even in the context of IFN-free DAA therapy, different HCV genotypes or subtypes can respond differently to the same DAA agent, leading to different resistance profiles [13]. Thus, it is imperative to clarify the current HCV genotype and subtype distribution in chronic hepatitis C patients in China.

In 2005, Lu et al. reported the HCV genotype distribution in China; they found that genotypes 1b and 2a were the dominant subtypes and genotypes 3 and 6 were limited to southern and southwestern China [6]. Since then, many studies of genotype/subtype distribution in local regions and specific populations have been reported [7–10]. Particularly recently, Li et al. reported the epidemiology of hepatitis C virus infection in highly endemic HBV areas [7]. Gu et al. conducted phylogenetic analyses of HCV in southern China, and revealed the constantly changing pattern of HCV genotypes in China over time [8]. Rao et al. reported the HCV genotype distribution in chronic hepatitis C patients in the Han ethnic population in China [10]. To provide a clear picture of the nationwide genotype distribution of HCV infection, we selected 23 hospitals from six geographic regions of China and enrolled 1012 naive chronic hepatitis C patients. Overall, the predominance of subtype 1b, followed by 2a, is in concordance with previous studies performed in different regions and different populations of China [7–10]. Previously, Lu et al. reported that genotype 6 accounted for 12.94 % of HCV infections in China [6]. In contrast, however, the frequency of this genotype in our study was much lower (2.58 %). We consider that this difference is due to patient selection, since more patients were enrolled from southern China in the Lu et al. study, where genotype 6 HCV infection is more prevalent than in other areas of China [8, 10, 14, 15].

Previous studies characterized genotypes 3 and 6 of HCV as geographically limited to western and southern China [6, 14, 15]. Recently Rong et al. reported that genotype 6a has spread from southern China and is now present nationwide [16]. The results were confirmed in this study, since we also found subtype 6a infection in eastern China, northern China, and northeastern China. However in this study, genotype 6n was also found in eastern China. Gentoypes 3a and 3b were found in northeastern, northern, and eastern China. Taken together, these results reflected that genotypes 3 and 6 have already spread nationwide, possibly due to economic reasons.

The greatest diversity of HCV subtypes was found in eastern China, which may be explained by the rapid economic development and high population mobility. Only 2 HCV subtypes were found in patients from central China, which may be related to the relatively small sample size and low population mobility in this area.

Our results support a correlation between HCV subtypes and routes of HCV transmission. More than 30 % of individuals with HCV subtypes 1b and 2a had a self-reported history of each blood transfusion and surgery; this was significantly higher than reports from genotype 3 patients. Among patients with a history of blood transfusion, 86.3 % (297/344) received blood before 1992; in this period, there was no screening of donated blood and blood products for HCV. These results indicate that subtypes 1b and 2a are more related to blood transfusion than genotype 3, and also partially explain why subtype 1b and 2a patients are generally older than genotype 3 patients when first diagnosed. Previous studies from other countries and regions reported that genotypes 6 and 3 are more frequent among IDU patients than in the general population [17–19]. Our current results are consistent with these reports, since more Chinese genotype 3 and 6 patients had a history of IDU when compared to genotype 1b and 2a patients. Although genotype 3 and 6 patients are younger than genotype 1b and 2a patients, IDU hepatitis C patients have higher risk of co-infection of HIV and HBV, which may accelerate disease progression. Therefore, special attention should be paid to these IDU patients.

## Conclusion

This study reveals the current HCV genotype and subtype distribution among Chinese chronic hepatitis C patients. At least four genotypes and seven subtypes were prevalent nationally. The distribution of HCV genotypes vary by geographic region, while subtypes 1b and 2a remain the most prevalent in China. Genotype 3 and 6 HCV infections are no longer restricted to southern and western China, but are now nationwide, and this altered distribution appears closely related to IDU.

## Materials and methods

### Study population

From June 2011 to March 2012, treatment-naive chronic hepatitis C patients were randomly enrolled from 23 hospitals in China. HCV infection had been confirmed (anti-HCV antibody and HCV RNA-positive) within 90 days prior to enrollment. Patients who had received anti-HCV therapy were excluded; no other exclusion criteria were applied. Patients were from 24 administrative units, which covered northeast China (Heilongjiang Province, Jilin Province, and Liaoning Province), northern China (Beijing Municipality, Tianjin Municipality, Hebei Province, Inner Mongolia Autonomous Region, and Shanxi Province), central China (Henan Province, Hubei Province, Hunan Province), eastern China (Anhui Province, Zhejiang Province, Shanghai Municipality, Jiangsu Province, Shandong Province, Jiangxi Province, and Fujian province), southern China (Guangdong Province), and western China (Sichuan Province, Xinjiang Uygur Autonomous Region, Qinghai Province, Shaanxi Province, and Gansu Province). Demographic information (gender, age, birthplace, etc.) and transmission risk factors (including history of blood transfusions, surgeries, invasive procedures (dental work, tattooing/piercing, etc.), or intravenous drug use (IDU), and family clustering of HCV infections) were collected from each patient with a face-to-face interview based on a questionnaire. Serum specimens were collected and sent to the central laboratory of the Beijing Ditan Hospital for study.

This study was approved by the ethics committee of Beijing Ditan Hospital of Capital Medical University. Written informed consent was obtained from all subjects when enrolled.

### HCV RNA extraction and reverse-transcription polymerase chain reaction (RT-PCR)

HCV RNA was extracted from 140 μL of each serum sample using the QIAmp Viral RNA Mini kit (Qiagen China Co., Ltd, Shanghai, China), followed by one-step RT-PCR assays with the One Step RNA PCR kit (AMV; TaKaRa Biotechnology Co. Ltd., Dalian, China). Outer sense and antisense primers derived from the NS5B and CORE/E1 region of the HCV genome were used for RT-PCR as described previously [20]. Nested PCR was performed using 10 μL of the RT-PCR product using GoTaq Colorless Master Mix (Promega Biotech Co Ltd., Beijing, China), and primers as specified previously [20]. The final PCR products were confirmed by 2 % agarose gel electrophoresis and visualization with ethidium bromide staining. Amplified products were sent to Beijing Centre for Physical and Chemical Analysis for sequencing with both the sense and antisense inner primers.

### HCV genotyping and phylogenetic analyses

The sequences of the core/E1 and NS5B regions were aligned and edited with reference sequences of each subtype using the CLUSTALW multiple alignment program in BioEdit software [21]. HCV subtype reference sequences were derived from the Los Alamos HCV database [22]. Phylogenetic analysis was performed using the neighbor-joining method using MEGA 4, and the Kimura two-parameter method was chosen to evaluate genetic distances [23]. The reliability of the phylogenetic clustering was evaluated using bootstrap analysis with 1000 replicates. A patient’s HCV subtype was determined when it was included in a defined cluster with given reference strains and with a bootstrap value above 70.

### Statistical analysis

Statistical data analyses were performed with SPSS 13.0. The continuous data were expressed as the mean ± standard deviation (SD) or as otherwise stated, and categorical variables in absolute numbers and percentages. The student’s *t*-test was used to compare the mean ages between patients infected with different HCV subtypes. The Pearson Chi-Square test was used to compare the ratio of risk factors between different genotypes. *p* values < 0.05 were considered statistically significant.

### Ethical considerations

This study was approved by the Ethics Committee of Beijing Ditan Hospital, Capital Medical University.

## Additional files

Additional file 1: Figure S1. Subtype 1a phylogeny estimated from NS5B region sequences (H77 positions: 8244–8713). Subtype 1b sequence AB049088 and subtype 2a sequence D00944 were used as outgroups. Black circles are Chinese isolates reported in other studies and white circles label reference sequences from outside China. Sequences without a circle were from this study. Additional file 2: Figure S2. Subtype 1b phylogeny estimated from NS5B region sequences (H77 positions: 8244–8713). Subtype 1a sequence NC 004102 and subtype 2a sequence D00944 were used as outgroups. Black circles are Chinese isolates reported in other studies and white circles label reference sequences from outside China. Sequences without a circle were from this study. Black triangles represent the locations of clusters, which is not showed in detail for shortage of space. Additional file 3: Figure S3. Subtype 2a phylogeny estimated from NS5B region sequences (H77 positions: 8244–8713). Subtype 1a sequence NC 004102 and subtype 2b sequence AB559564 were used as outgroups. Black circles are Chinese isolates reported in other studies and white circles label reference sequences from outside China. Sequences without a circle were from this study. Black triangle represents the location of the cluster, which is not showed in detail for shortage of space. Additional file 4: Figure S4. Subtype 3a phylogeny estimated from NS5B region sequences (H77 positions: 8244–8713). Subtype 1a sequence NC 004102 and subtype 3b sequence D49374 were used as outgroups. Black circles are Chinese isolates reported in other studies and white circles label reference sequences from outside China. Sequences without a circle were from this study. Additional file 5: Figure S5. Subtype 3b phylogeny estimated from NS5B region sequences (H77 positions: 8244–8713). Subtype 1a sequence NC 004102 and subtype 3a sequence EU710463 were used as outgroups. Black circles are Chinese isolates reported in other studies and white circles label reference sequences from outside China. Sequences without a circle were from this study. Additional file 6: Figure S6. Subtype 6a phylogeny estimated from CORE/E1 region sequences (H77 positions: 834–1318). Subtype 1a sequence NC 004102 and subtype 6b sequence D37841 were used as outgroups. Black circles are Chinese isolates reported in other studies and white circles label reference sequences from outside China. Sequences without a circle were from this study. Additional file 7: Figure S7. Subtype 6n phylogeny estimated from CORE/E1 region sequences (H77 positions: 834–1318). Subtype 1a sequence NC 004102 and subtype 6a sequence Y12083 were used as outgroups. Black circles are Chinese isolates reported in other studies and white circles label reference sequences from outside China. Sequences without a circle were from this study.

## Abbreviations

HCV: Hepatitis C virus
IDU: Intravenous drug use
RT-PCR: Reverse-transcription polymerase chain reaction
DAA: Direct-acting antivirals
