# Supplementary figures and images for: Hepatitis C virus genotype and subtype distribution in Chinese chronic hepatitis C patients: nationwide spread of HCV genotypes 3 and 6
Source: Virol J. 2015 Jul 25;12:109. doi: 10.1186/s12985-015-0341-1 (PMC4513753; doi:10.1186/s12985-015-0341-1)

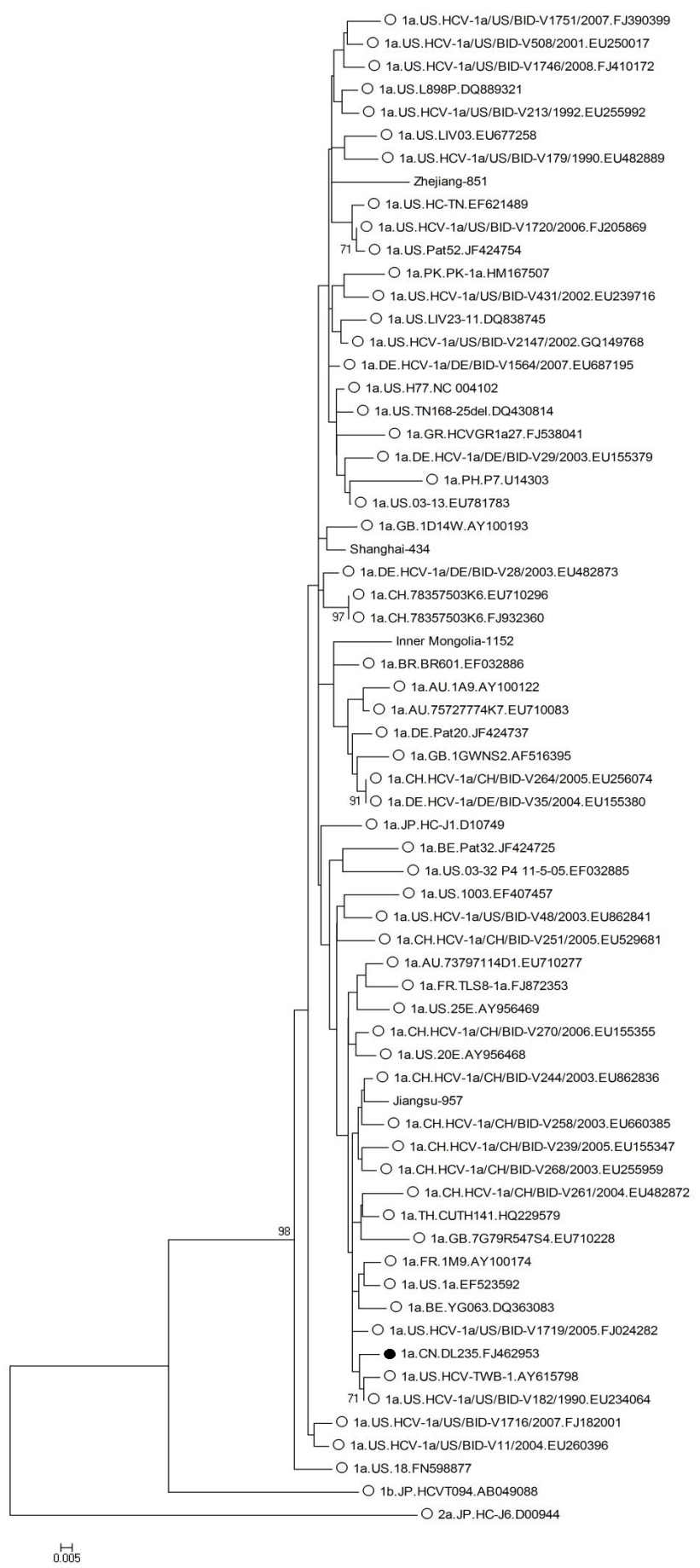


Additional file 1: Figure S1

Supplement: Additional file 1: Figure S1. — Subtype 1a phylogeny estimated from NS5B region sequences (H77 positions: 8244–8713). Subtype 1b sequence AB049088 and subtype 2a sequence D00944 were used as outgroups. Black circles are Chinese isolates reported in other studies and white circles label reference sequences from outside China. Sequences without a circle were from this study. [file 12985_2015_341_MOESM1_ESM.doc]

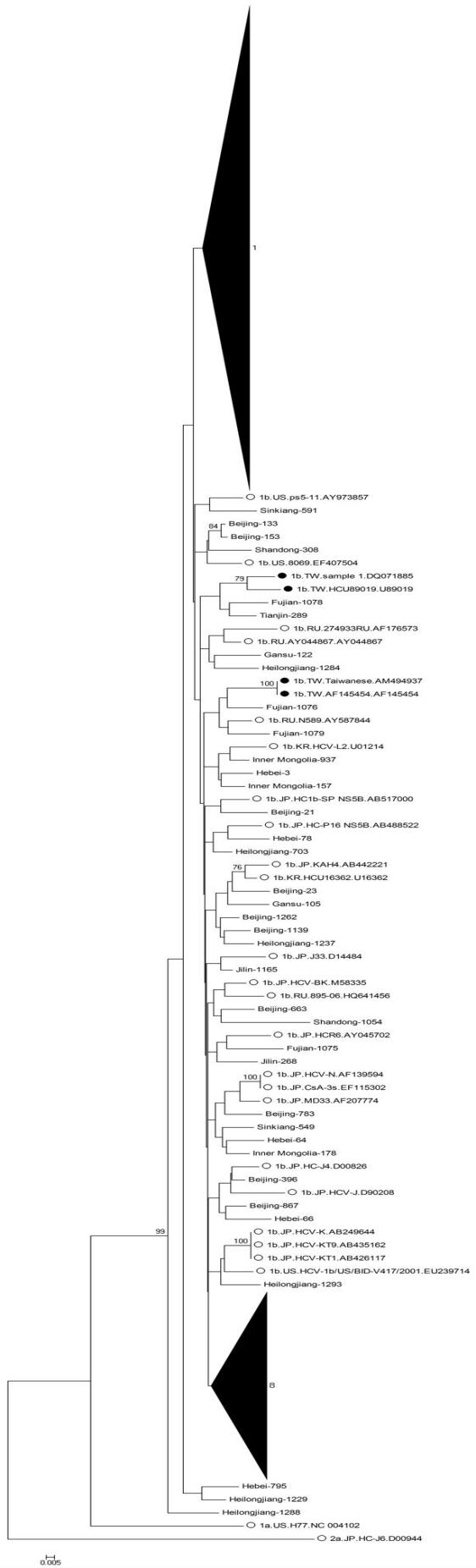


Additional file 2: Figure S2

Supplement: Additional file 2: Figure S2. — Subtype 1b phylogeny estimated from NS5B region sequences (H77 positions: 8244–8713). Subtype 1a sequence NC 004102 and subtype 2a sequence D00944 were used as outgroups. Black circles are Chinese isolates reported in other studies and white circles label reference sequences from outside China. Sequences without a circle were from this study. Black triangles represent the locations of clusters, which is not showed in detail for shortage of space. [file 12985_2015_341_MOESM2_ESM.doc]

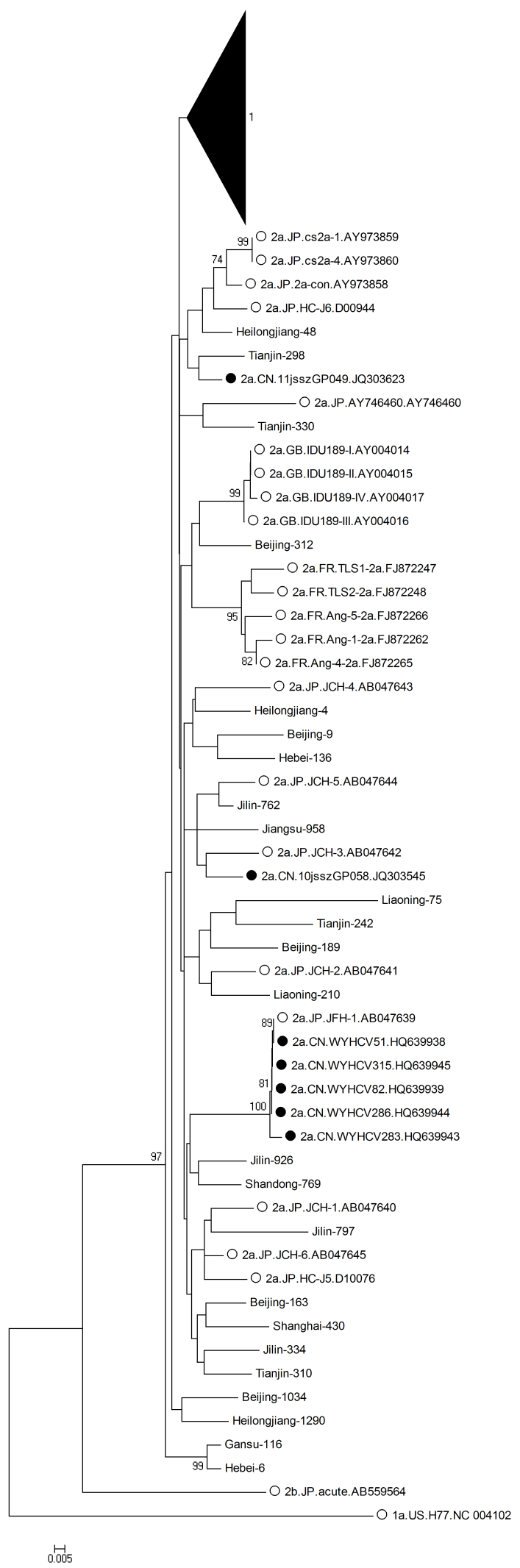


Additional file 3: Figure S3

Supplement: Additional file 3: Figure S3. — Subtype 2a phylogeny estimated from NS5B region sequences (H77 positions: 8244–8713). Subtype 1a sequence NC 004102 and subtype 2b sequence AB559564 were used as outgroups. Black circles are Chinese isolates reported in other studies and white circles label reference sequences from outside China. Sequences without a circle were from this study. Black triangle represents the location of the cluster, which is not showed in detail for shortage of space. [file 12985_2015_341_MOESM3_ESM.doc]

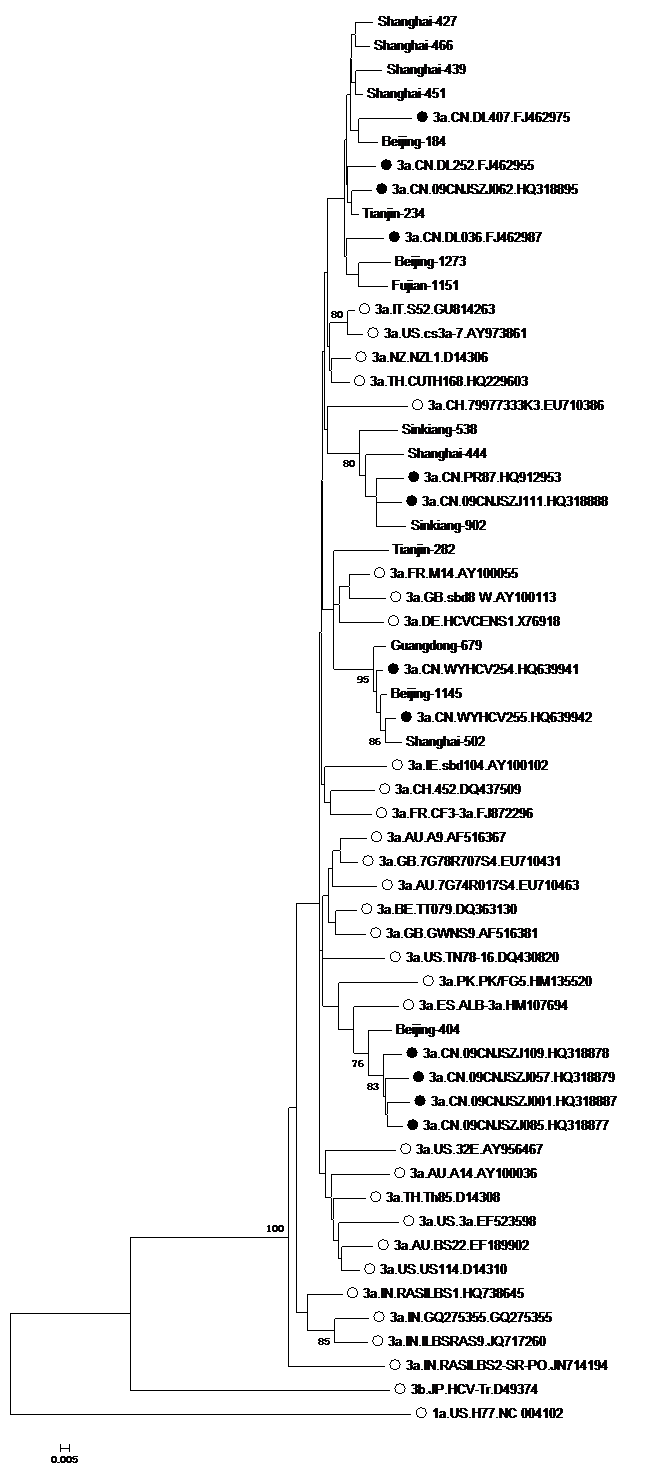


Additional file 4: Figure S4.

Supplement: Additional file 4: Figure S4. — Subtype 3a phylogeny estimated from NS5B region sequences (H77 positions: 8244–8713). Subtype 1a sequence NC 004102 and subtype 3b sequence D49374 were used as outgroups. Black circles are Chinese isolates reported in other studies and white circles label reference sequences from outside China. Sequences without a circle were from this study. [file 12985_2015_341_MOESM4_ESM.doc]

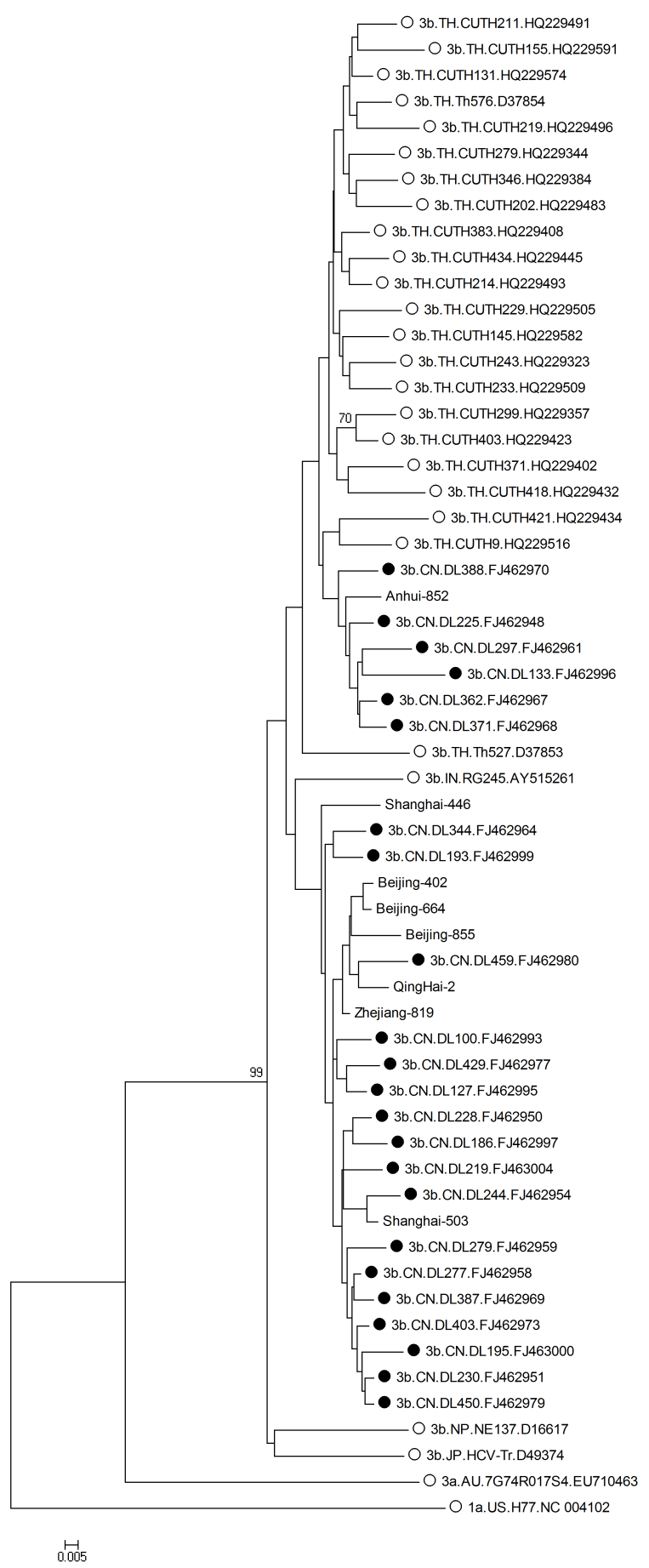


Additional file 5: Figure S5.

Supplement: Additional file 5: Figure S5. — Subtype 3b phylogeny estimated from NS5B region sequences (H77 positions: 8244–8713). Subtype 1a sequence NC 004102 and subtype 3a sequence EU710463 were used as outgroups. Black circles are Chinese isolates reported in other studies and white circles label reference sequences from outside China. Sequences without a circle were from this study. [file 12985_2015_341_MOESM5_ESM.doc]

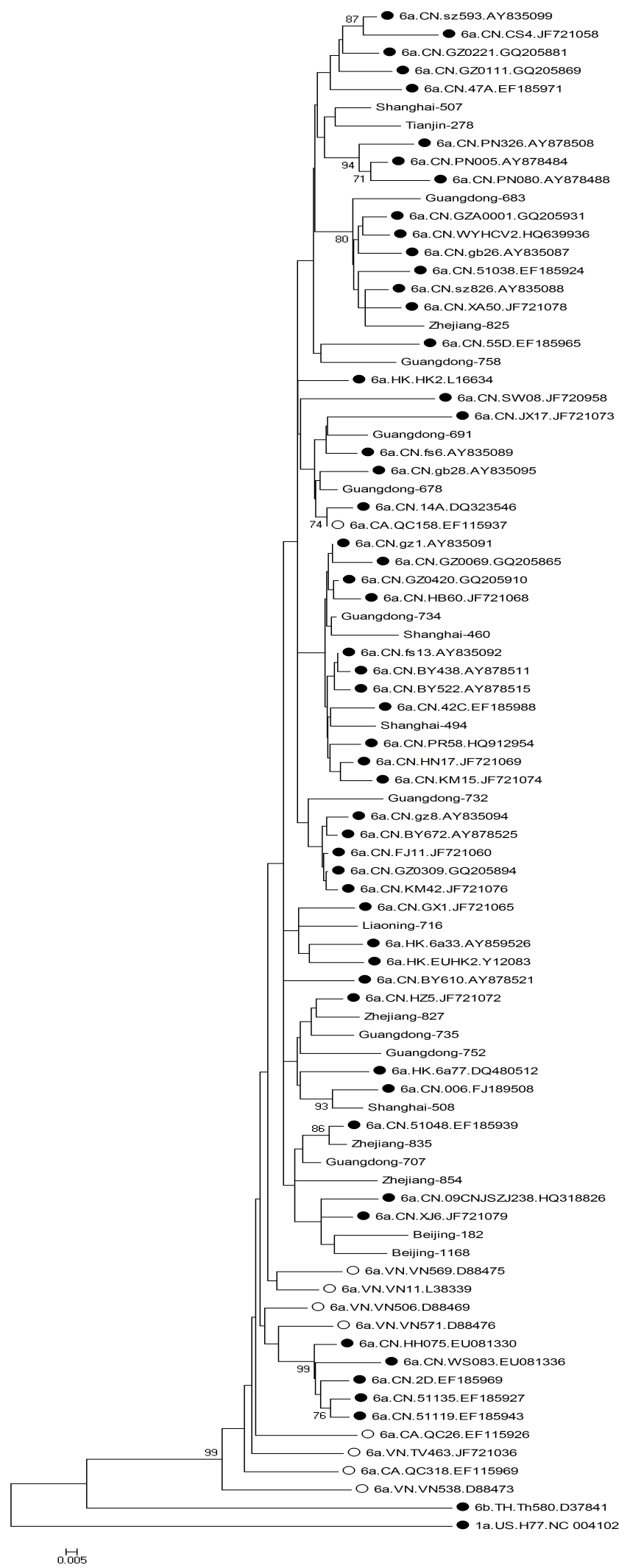


Additional file 6: Figure S6.

Supplement: Additional file 6: Figure S6. — Subtype 6a phylogeny estimated from CORE/E1 region sequences (H77 positions: 834–1318). Subtype 1a sequence NC 004102 and subtype 6b sequence D37841 were used as outgroups. Black circles are Chinese isolates reported in other studies and white circles label reference sequences from outside China. Sequences without a circle were from this study. [file 12985_2015_341_MOESM6_ESM.doc]

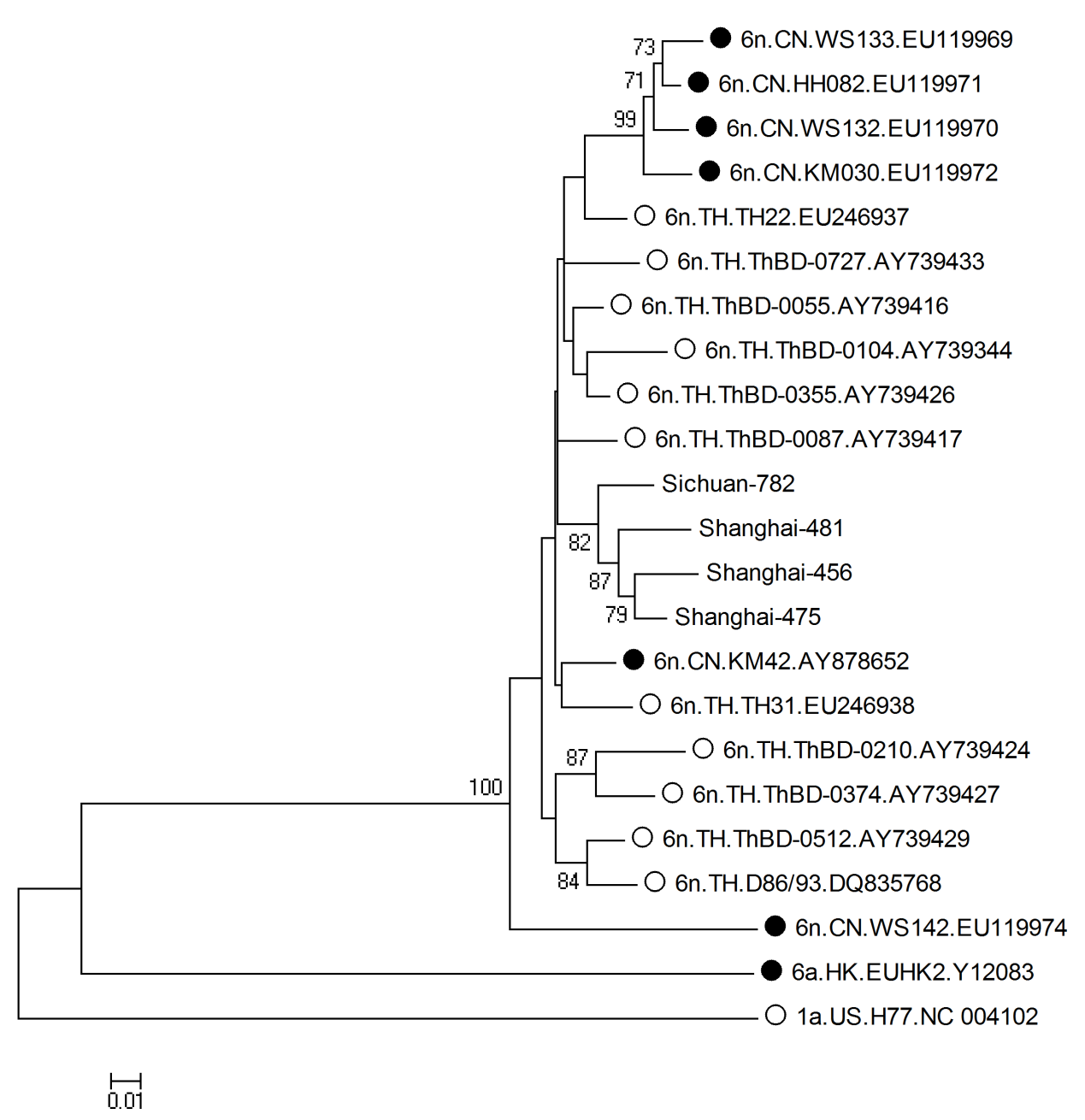


Additional file 7: Figure S7.

Supplement: Additional file 7: Figure S7. — Subtype 6n phylogeny estimated from CORE/E1 region sequences (H77 positions: 834–1318). Subtype 1a sequence NC 004102 and subtype 6a sequence Y12083 were used as outgroups. Black circles are Chinese isolates reported in other studies and white circles label reference sequences from outside China. Sequences without a circle were from this study. [file 12985_2015_341_MOESM7_ESM.doc]
